# Guidance for Healthy and More Climate-Friendly Diets in Nursing Homes—Scenario Analysis Based on a Municipality’s Food Procurement

**DOI:** 10.3390/nu13124525

**Published:** 2021-12-17

**Authors:** Anne Dahl Lassen, Matilda Nordman, Lene Møller Christensen, Anne Marie Beck, Ellen Trolle

**Affiliations:** 1 Division of Food Technology, National Food Institute, Technical University of Denmark, DK-2800 Kgs, Denmark; matnor@food.dtu.dk (M.N.); lmch@food.dtu.dk (L.M.C.); eltr@food.dtu.dk (E.T.); 2 Dietetic and Nutritional Research Unit, Herlev and Gentofte University Hospital, DK-2730 Herlev, Denmark; Anne.Marie.Beck@regionh.dk; 3 Faculty of Health, Institute of Nutrition and Nursing, University College Copenhagen, DK-1799 Copenhagen, Denmark

**Keywords:** public food service, dietary guidelines, sustainable food procurement, older adults, protein- and energy-dense diet

## Abstract

Reducing the climate impact of food provided for residents in nursing homes is challenging, as the diets for older, frail adults must be high in protein content and energy density while at the same time ensuring that the meals are palatable and recognizable. This study aimed at providing guidance on healthy and more climate-friendly diets for nursing homes in the City of Copenhagen. The goal was to decrease greenhouse gas emissions (GHGE) by at least 25% while at the same time providing nutritionally adequate and recognizable menus. First, food purchase data were compiled with datasets matching each food item to a proxy food item and then to databases containing GHGE and nutrient information. Secondly, two diet scenarios were modelled based on current procurement practices, i.e., an energy- and protein-dense diet and a standard protein-dense diet, and converted into guidelines for menu planning. The diets contained less total meat, especially beef, and significantly more pulses, nuts and seeds in order to increase protein content according to recommendations for older adults. Finally, a combined scenario was calculated to reflect the joint climate impact reduction. This kind of innovation in food procurement is required in order to achieve the necessary transition to a sustainable food system.

## 1. Introduction

The current food system is responsible for approximately a third of global anthropogenic greenhouse gas emissions (GHGE) [1] and accounts for a significant proportion of the planet’s environmental challenge, including nitrogen and phosphorus emissions, land use, freshwater use, biodiversity loss and pesticide emissions [2]. There is a need to reduce these impacts at all stages of the food supply chain from primary production and processing to retailers, food service, consumers’ dietary habits and food waste [2,3].

In terms of providing industry with incentives for developing environmentally sustainable products and services and thereby advancing sustainable food development, governments and municipalities have a unique opportunity to lead by example through the implementation of sustainable public food procurement [4,5,6]. The European Union Green Public Procurement program provides a voluntary framework to encourage public bodies to procure sustainable goods and services. This includes specifications with the goal of increasing the consumption of pulses, vegetables, fruits, whole grains and nuts whilst having the same recommended nutrient intake for the clients [4]. In addition, public procurement of organic food products has been widely applied and called for at different governmental levels in the EU in order to expand organic farmland [7] In 2021, the WHO [6] submitted an action framework to support the development and implementation of food procurement and related service policies for a healthy diet that also incorporates sustainable actions via, e.g., sustainable purchasing of local and seasonal food.

Many municipalities and cities have set targets for how they will work to ensure healthy and sustainable procurement and meals [8]. This applies, for example, to the City of Copenhagen that has set a target to reduce GHGE from public meals by 25% by 2025 compared with levels from 2018 while ensuring nutritional requirements, culinary quality and keeping the organic food purchase at a high level [9].

Thus far, guidance on healthy, sustainable menus at nursing homes is very limited. Municipalities provide meals to vulnerable groups of the population, including institutionalized older adults. Older age is associated with a decreased food intake and a higher prevalence of malnutrition [10]. Low energy intake and nutrient deficiencies are considered to be the main causes of malnutrition in institutionalized people [11]. Hence, active support and focus on food intake to ensure good nutritional status is needed, including routine screening for (risk of) malnutrition in older adults in nursing homes and in the community at admission/initial contact [12]. Furthermore, a focus on providing nutritious main meals, in-between meals, as well as nourishing drinks that fit the target groups’ needs and food preferences is of utmost priority [12,13,14]. Protein and energy enriched food-service meals have been shown to increase energy and protein intake and improve the nutritional status of nutritionally at-risk older adults [15].

The current European recommendation of protein intake for older adults is a minimum of 1.0 g protein per kg body weight per day [12]. The Nordic Nutrition Recommendations (NNR) recommend that for dietary planning purposes in the elderly, a suitable target for protein intake should be about 1.2 g protein per kg body weight per day [16]. For older adults with acute or chronic illness, daily amounts of 1.2–1.5 g protein per kg body weight per day have been suggested [12,17]. This is relevant for a significant part of the residents at nursing homes [18,19,20]. An insufficient energy intake further increases the protein requirement. Thus, it is important to ensure not only an adequate intake of protein but also an appropriate intake of energy [12]. The Danish official recommendations regarding the food served in nursing homes recommend that an energy- and protein-dense menu should contain about 50% of energy from fat and 18% of energy from protein [14]. This corresponds to up to about 1.4 g protein per kg body weight per day, assuming an average body weight of 67 kg and average energy requirement of 8.9 MJ [16]. In addition, it is recommended that the energy- and protein-dense diet is supplemented by a daily multivitamin and mineral supplement [14].

While the energy- and protein-dense diet should be the preferred menu served at nursing homes, a standard protein-dense diet with lower fat content but likewise 18% energy (E%) from protein should be made available for residents without nutritional problems [16]. The challenge is how to ensure a high protein and energy density of the diets of older adults while at the same time lower the climate impact [21] and maintain that the meals are recognizable [22]. Based on the analysis of 47 main meal recipes from five meal service producers, Saxe et al. [23] suggest that the most important strategy for reducing the environmental impact of main meals is to reduce the number of meals containing veal and beef. Additionally, combining plant-based proteins with animal-based proteins could be an important strategy. Verzola et al. [24] argue that combining plant- and animal-based proteins in sufficient amounts can activate muscle protein anabolism in a similar way to high-quality proteins and that this is the case also in older adults. Finally, the focus should be on minimizing food waste, which has been described to be at high levels in nursing homes [25,26]. High food waste means that important nutrients are not used for human consumption but instead go to waste.

The overall aim of this study was to provide guidance for a 25% reduction in GHGE for food purchased by the nursing homes in the City of Copenhagen while simultaneously providing nutritionally adequate, affordable and recognizable meals. More specifically, the aim was to investigate scenarios for two dietary patterns, i.e., an optimized energy- and protein-dense diet (Scenario 1) and an optimized standard protein-dense diet (Scenario 2), respectively, as well as a combined scenario (Scenario 3) to provide the climate reduction potential of the total food purchase. Finally, the study aimed to translate the diets to guidelines for menu planning, including rules of thumb for the number of servings for each main food group over a one-week period of time.

## 2. Materials and Methods

### 2.1. Data Description and Compilation

Three overall datasets were combined using Excel Power Pivot. A food purchase dataset for the year of 2018 was compiled with a dataset matching each unique food item to estimates on edible amounts (depending on processing level, etc.) and to a proxy food item present in The Danish Food Composition Database [27]. Then data were compiled with nutritional composition data as well as climate impact data on the proxy food items. The method of data compiling is described in detail in a previous paper [28], focusing on scenario analysis of the City of Copenhagen’s food purchase to improve nutritional quality and lower carbon emission for the food provided to child-care centres.

In short, food purchase data were retrieved from the main wholesale food supplier for the year 2018. It contained amounts of foods purchased in kilogram, price per weight and info on certified organic products. Purchase data from smaller suppliers providing single food categories (e.g., fresh fish) were also retrieved, where purchase data from smaller suppliers offering a variety of food categories were excluded in order to simplify the calculation. Based on the total expenditure on food procurement in the municipality, the dataset was estimated to comprise 88% of the total food purchased and was judged to be reasonably representative of the full procurement on the food group level [28].

Nutritional data were retrieved from The Danish Food Composition Database [27]. The data were updated with newer analyses and checked and updated for missing data, as described by Lassen et al. [28]

Climate impact data were provided from the World Resources Institute’s Cool Food Pledge food purchasing tracking sheet [29] with a few modifications [28]. The sheet includes default weighted European average factors for GHGEs by food category from the whole agricultural supply chains as well as global-average factors for carbon opportunity costs (Metric 4) [29]. Estimation of climate impact was based on the edible amounts, e.g., excluding bones, etc., in line with the World Resources Institute data (boneless equivalent).

### 2.2. Baseline and Diet Scenario Modelling

**Figure 1:**
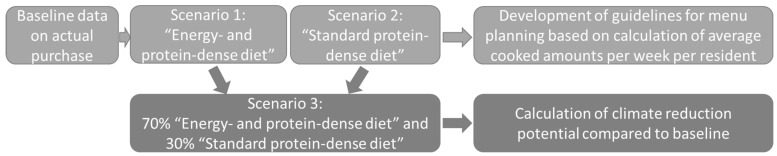
Overview of the scenarios modelled and their usage for guideline development and calculation of the joint reduction potential compared with the baseline.

Figure 1 gives an overview of the scenarios modelled and their usages. Two dietary patterns were modelled (Scenarios 1 and 2) based on actual purchase data for nursing homes preparing all meals in-house, as data on these nursing homes represent a whole day’s dietary pattern (baseline data). Moreover, a combined scenario (Scenario 3) was modelled based on the estimated serving frequency of the two dietary patterns.

Scenarios 1 and 2 included optimizing two diets for older adults, i.e., an energy- and protein-dense diet and a standard protein-dense diet, respectively, to be in accordance with Danish recommendations for dietary planning among institutionalized people [14]. This included a protein content of 18 E% for both scenarios and a fat content of approximately 50 E% for the energy- and protein-dense diet and content of no more than 33 E% from total fat (maximum of 10 E% from saturated fat) for the standard protein-dense diet. Nutritional outcomes included macronutrient distribution as well as added sugar, dietary fibre and n-3 fatty acids content. Further, the scenarios were modelled to reach the combined GHGE reduction target, keep additional economic cost within +5%, and at the same time, making as few changes as possible to accommodate current food preferences.

The modelling was based on main food groups and subgroups as described by Lassen et al. [28]. Food groups included starch-rich foods (potatoes, bread, pasta, rice, cereals and oat products), fruits and berries (including juice), vegetables (dark green and red-orange vegetables, green peas and other vegetables), plant-based protein-rich foods (pulses, nuts and seeds); meat and eggs (beef, lamb, pork, poultry and eggs), fish and seafood (subdivided into three climate impact categories), dairy products (milk and fermented milk according to fat content, cheese and other dairy products), animal-based and plant-based fats (butter, oils, fatty sauces and dressings), discretionary foods and beverages (sugar-rich foods, savoury snacks and sweetened beverages), and condiments and seasoning (spices and dips/sauces). In the present study, pulses were separated into whole pulses and pulse flour to underline different ways of using pulses in meal preparation. Pulses are defined as a subgroup of legumes, including chickpeas, cowpeas, dry beans, dry peas, and lentils [30]. Dried soybean was used as a proxy food for the category pulse flour, which may also include other plant-based protein-rich flours and powders and in smaller amounts animal-based protein powders.

The proportion of food items within the food groups reflected the current food purchase practice for nursing homes to take into account the present food preferences of the residents. Modelling included both substitution of foods within food groups, i.e., choosing the more environmentally friendly products—and in some cases the more protein-rich foods—within the different food groups, and substitution of foods between food groups, e.g., switching meat and animal fat with plant-based protein-rich foods and fats. Further, extra protein-rich plant products, e.g., pulses, nuts and seeds, were included to increase the total protein content of the diets.

### 2.3. Development of Guidance for Menu Planning

Scenarios 1 and 2 provided the basis for the guideline development. First, quantities of purchased food per 10 MJ were converted to cooked quantities for a 9 MJ per day (2100 kcal) diet over a week. Then, rules of thumb for frequency of serving for each main food group were calculated from estimated average portion sizes modified from the digital Dietary Reference Manual [31], which is based on the Danish official recommendations regarding the food served in Danish institutions [14]. Frequency of serving was communicated for hot meals and other meals separately in order to cover different types of nursing homes with or without in-house hot meal production and catering services delivering hot meals to nursing homes and home residing individuals (“meals on wheels”).

The usefulness and feasibility of the guidance for menu planning were discussed in dialogue meetings with key actors consisting of gastronomic consultants for the municipality, key persons in the municipality and an academic expert in nutritional requirements in geriatrics (A.M.B.). Kitchen staff at three nursing home centres with in-house meal production pilot-tested the guidelines in practice and were interviewed about their experiences. Furthermore, two nursing homes where the hot meals were provided by an internal catering service were introduced to and interviewed about the guidelines. Finally, additional dialogue meetings were established with the internal catering service supplying hot meals to nursing homes and “meals on wheels” to discuss the feasibility of implementing the developed guidelines for menu planning.

### 2.4. Calculation of Climate Reduction Potential

Scenario 3 provided the basis for the calculation of the joint climate reduction potential arising from a change in dietary composition. In the present study, it is estimated that about 70% of the diets provided should be energy- and protein-dense and the rest standard protein-dense. This is based on a systematic review and meta-analysis estimating that, on average, two-thirds of nursing home residents either suffer from malnutrition or are at risk of malnutrition [32]. The 25% reduction goal was based on GHGE from Metric 2 (total agricultural supply chains) together with Metric 4 (global-average factors for carbon opportunity costs).

## 3. Results

### 3.1. Background Information

The total dataset for the food procurement in the municipality contained approximately 8700 tons of food and beverages. Included in the present study was food purchased by nursing homes with in-house meal production, constituting 23% of the total amount of food purchased in the municipality. The share of registered organic food procurement (percentage of total purchased weight) was 78% for these nursing homes.

Furthermore, within the area of care for older adults, nursing homes where the hot meals are provided by a catering company accounted for 5% of the purchased food and the municipality’s catering service supplying “meals on wheels” and hot meals to nursing homes accounted for 7% of the purchased food.

### 3.2. Baseline and Diet Scenarios

Diet scenario models were developed and described in terms of g per 10 MJ for each food group. The models were slightly modified based on the dialogue meetings and pilot tests with the nursing homes. This resulted in a small increase in the amount of butter and cheese as it was considered important for the residents to be able to have butter and cheese on the bread. In addition, the amount of nuts was reduced, as many of the residents are not capable of eating whole nuts. Table 1 shows the amounts of foods at baseline and in the final scenarios.

Compared with the baseline, the total amount of meat was reduced in all the scenarios with about one-quarter, especially owing to a lower content of beef, veal and lamb. In addition, the amount of cheese was lowered by about one-third in all scenarios. On the other hand, the amount of fish and eggs was increased. The distribution of types of seafood (fish and shellfish) was changed toward decreasing seafood with a higher climate impact, i.e., from 13% to 5% seafood with the highest climate impact (e.g., shrimps).

The amount of fatty dairy foods (cream etc.) and animal fat (butter) was decreased by a little more than one-third in Scenario 1 in order to lower climate impact and at the same time lower the amount of saturated fat in favour of vegetable oil that contains larger amounts of mono- and polyunsaturated fatty acids.

The amounts of plant-based protein-rich foods were increased significantly in the scenarios to compensate for the lower meat content but also to increase the total protein content compared with the baseline. The distribution of different types of vegetables was changed in all scenarios toward relatively more dark green vegetables and green peas, and the distribution of bread and cereals was changed towards relatively more oatmeal and a little quinoa to optimize protein content.

At baseline, the percentage of energy (E%) from protein was found to be 13 in the food purchased and therefore lower than recommended (Table 2). The percentage of energy from fat was 47 and thereby close to the recommended 50 E% for an energy- and protein-dense diet. The energy contribution from saturated fatty acids was high at the baseline and for the energy- and protein-dense diet but was within the maximum recommended level of 10 E% for the standard protein-dense diet. Fibre content was higher in all scenarios compared with the baseline (18 g per 10 MJ at baseline and 23 g per 10 MJ in the energy- and protein-dense diet).

### 3.3. Guidelines for Menu Planning

Table 3 shows the main guidelines for menu planning, including suggestions for frequency of serving and calculated average cooked amounts for a week (7 days). 

The guidelines suggest a maximum amount of 500 g cooked meat weekly per resident, including no more than 80–85 g beef, veal or lamb per week. As meat is part of many traditional Danish dishes consumed by older adults, the possibility of serving five weekly hot meals with meat was prioritized in the guidelines for menu planning (including a maximum of one weekly meal with beef, veal or lamb). Instead of further reducing the frequency of serving meat at the hot meals, portion sizes were decreased to a maximum of 75 g meat per meal on average in order to reduce the total amount of meat on the menu. This amount should be supplemented with plant-based protein-rich foods at the meals, e.g., pulses mixed with meat, blended in soup, sauce, in bread, etc. Fish and vegetarian dishes should be served at least once a week each as a hot meal and daily as part of the cold meal (300 g cooked fish/week and 240 g egg/week). In Denmark, the cold meal typically consists of 3–4 open sandwiches. 

Guidelines also emphasize regularly serving pulses distributed over the day (approximately 300 g cooked per week), as well as nuts and seeds (approximately 125 and 100 g/week, respectively). Additionally, 300 g pulse flour (or other protein-rich powders) for the energy- and protein-dense diet and 100 g pulse flour for the standard protein-dense diet should be included. 

Compared with the standard protein-dense diet, the energy- and protein-dense diet contained significantly fewer starchy foods, fruits and vegetables in order to keep the volume of the meals low and increase energy density. On the other hand, the amount of fatty products, including fatty dairy products, was much higher in the energy- and protein-dense diet compared with the standard protein-dense diet. For both diets, the use of plant-based fats was emphasized.

### 3.4. Climate Reduction Potential and Economic Costs

Table 4 shows that an estimated 22% reduction in GHGE from total agricultural supply chains (Metric 2) can be achieved by changing to the combined Scenario 3. Estimated reductions are greater when including potential carbon opportunity costs (COC) (Metric 2 + 4), approximately 26%.

Economic cost of the Combined Scenario 3 was estimated to be +5% compared with the baseline.

## 4. Discussion

Reducing the climate impact of food provided for residents in nursing homes is challenging, as the diets for older adults should be high in protein content while at the same time ensuring that the meals are palatable and recognizable. Through scenario modelling based on actual purchase data from the City of Copenhagen, this paper demonstrated how the food purchase could be changed to improve nutritional content in order to meet dietary recommendations on protein content and other nutrients and simultaneously decrease climate impact by approximately 25% in food purchased by nursing homes.

The scenarios included both an energy- and protein-dense diet directed at residents suffering from malnutrition or at risk of malnutrition and a standard protein-dense diet directed at residents with a normal appetite. Reduction in the climate impact was achieved by both substitution of foods within food groups, i.e., choosing the more environmentally friendly products and/or the more protein-rich foods within the different food groups, as well as substitution of foods between food groups, e.g., switching meat and animal fat with plant-based foods and fats. Further, extra protein-rich plant-based products, such as pulses, nuts and seeds, were included to increase the total protein content of the diets compared with the baseline.

### 4.1. Baseline Data

Results revealed that fat content was consistent with recommendations for older adults at risk of malnutrition [14] when calculated based on the purchased food, whereas the content of protein was lower than recommended. Results are comparable to a Danish cross-sectional study analyzing procurement data for 58 nursing homes with in-house food production representing different regions and suppliers and having different levels of organic meal procurement [26]. Similar to the present study, fat content was calculated to be 47–48 E% among kitchens with an organic share of more than 70%, and the protein content was 13–14 E%, compared with 13 E% in the current study. The same picture with regard to protein content was seen in an older study from Beck and Hansen [33], where the macronutrient composition of different whole-day menus from 10 kitchens servicing older adults were chemically analysed. Furthermore, an Australian cross-sectional study among care facilities revealed that foods provided to the residents did not always meet nutritional requirements, with protein intakes frequently below recommended levels [34,35], indicating that low protein content is a general issue for meals served to older adults.

The level of saturated fat was found to be high (21 E%) due to a high amount of total fat and a high proportion of purchased butter, high-fat dairy products, cheeses and meat. The Finnish food recommendation for older adults states that fats obtained from food should mainly be soft, unsaturated fats. Hard fats should account for less than one-third of total fat intake [36]. In the present study, animal-based fats, mainly butter and other butter-based spreads well exceeded the content of plant-based fats in the purchased food.

At baseline, more than a quarter (28%) of the total meat was beef, veal or lamb, having a high climate impact. This corresponds to a study among five Danish kitchens, showing that the main hot meals served to the senior citizens were composed of 20% beef [23].

In the present study, the fibre content of the purchased food was found to be 16 g per 9 MJ (2100 kcal per day). The European Society for Clinical Nutrition and Metabolism emphasizes the importance of an adequate intake of dietary fibre, since dietary fibre contributes to normal bowel function, and states that daily amounts of 25 g can be regarded as a guiding value for older adults [12].

### 4.2. Diet Scenario and Guidelines

The amount of meat, fish, eggs and cheese were modelled to the same level in both Scenarios 1 and 2, as protein requirements for both healthy and frail older adults are the same. Compared with the baseline, the total amount of meat was reduced to no more than 500 g cooked meat per week per resident, with especially lower amounts of beef, veal and lamb. This corresponds to serving beef, veal and lamb maximum once a week in hot meals compared with the current practice of around twice a week. However, the amount of meat is not as low as the Danish dietary recommendations for the general population [37], including the healthy population of older adults [38]. On the other hand, the amount of plant-based protein-rich foods was increased considerably. More pulse flour was included in the energy- and protein-dense diet to compensate for lower cereal intake. This means that pulses should be included in meals every day—both in one of the main meals and in the in-between meals. Pulses and nuts are rich in many different nutrients besides protein, including magnesium. Poor magnesium status has been associated with several negative outcomes in older adults [39]. A higher amount of plant-based protein sources, such as pulses, would also benefit dietary fibre content. A study found that the administration of an oral hyperprotein supplement with fibre in aged subjects who were undernourished or at risk of malnutrition appeared to have no adverse effects on appetite or energy intake but, on the contrary, improved both their nutritional status and their intestinal habits [40].

Guyomarc’h et al. [41] argue that animal/plant mixes enable a reduction in animal protein consumption while preserving amino acid and micronutrient intakes, and Liu et al. [42] showed that in healthy elderly individuals fed with reconstituted protein meals, their post-prandial amino acid plasma availability was more balanced when milk, whey, soybean and pea proteins were ingested together compared with single protein sources. Further, several long-term studies indicate that a higher protein intake has favourable effects on muscle mass and function in older subjects and that plant-protein sources at low protein intakes have lower ability to stimulate protein synthesis in muscle and to cause muscle mass gain as compared with animal proteins. However, the gap between the anabolic effects of plant- and animal-based proteins can be filled with an adequate plant-protein intake (1.1–1.2 g/kg body weight) [24].

In the present study, the focus was also on using the more protein-rich foods within food groups, for example, oatmeal. Oats have one of the highest protein content among cereals and is a rich source of bioactive compounds [43].

### 4.3. Implementation and Future Directions

Little has been reported on practical food preparation and cooking strategies for lowering the climate impact of the meals for older adults and the implementation on a routine basis. While meal familiarity must be taken into account when offering food targeted to older adults [44], some novel culinary “twists” may be acceptable and could even potentially stimulate food intake by preventing boredom [45]. Partial meat replacement by integration of plant-based ingredients into meat dishes has been proven as a successful and consumer-accepted strategy [46]. Argel et al. found that adding pulses (lentil, chickpea, pea, and bean) to burgers was feasible with a reduction in the pork meat in the product by up to 44% [47]. In addition, the integration of pulses in a cereal product has been proven successful. Partial replacement of wheat flour with pulses might enhance total protein content of bread but also the protein profile [48]. Enrichment of cereal flour with pulses has the potential to improve the nutritional quality of bread, and porridge, etc., since there is an overall increase in the protein content of the composite food and a better amino acid balance due to the contribution of lysine by legumes and of methionine by cereal [49]. Importantly, pulses need to be prepared in specific ways to destroy lectins [50]. In addition, pulses, nuts and seeds and products such as tahini and peanut butter can be integrated into other parts of the meals, e.g., in dressings, sauces, soups, cakes and in-between meals and drinks.

It is essential to increase the protein content of each meal [51]. The protein intake pattern across the day, in addition to the total protein intake, has been suggested as an important factor for protein turnover and muscle mass [52]. Wendin et al. argue that it is possible to produce highly liked energy- and protein-enriched in-between meal products and drinks designed for older adults [53]. For example, in-between meals offered as “finger foods”—foods that can be easily eaten with fingers—can increase the pleasure of eating, increase food consumption and improve autonomy among older adults living with frailty and dementia [54]. Future studies should explore the possibility and acceptability of using more plant-based products in the in-between enriched meals and drinks. In addition, a retrospective analysis of previously published work showed that a higher relative (to body weight) protein intake from a main meal was required to stimulate the same myofibrillar protein synthetic response in healthy older adults compared to younger adults [55].

Importantly, changes in menus need to be communicated to the care staff, as they have a significant role in aligning the meals with the needs of those in their care [56] as well as providing time and support at the meals. Furthermore, they should be involved in reducing serving and plate food waste, which is found to be high in Danish nursing homes [25,26].

### 4.4. Strengths and Limitations

The present study has several strengths. To our knowledge, it is the first study to examine a municipality’s nursing home food purchase matching data for each food item with data on GHGE and nutritional quality. Diet modelling was based on actual purchases to reflect current preferences and practices as close as possible. An additional strength is that data were converted to guidelines for menu planning in dialogue with key actors in order to implement the guidance in the City of Copenhagen.

The method has low participant burden and is not subject to social desirability bias. However, the method is best suited to be used when there is only one or a few food suppliers. In addition, it was assumed that most food is provided through the facility’s food service and that most residents have limited access to externally prepared foods.

A limitation is that it is not possible to separate food used for different purposes, e.g., for older adults with different dietary requirements. Additionally, the data represent foods purchased, not the actual dietary intake. Edible waste may be relatively higher for some types of foods, e.g., extra potatoes and dessert may be provided by the kitchen in order to ensure sufficiency of food. In the present study, estimated unavoidable food losses (i.e., inedible parts) were accounted for in the calculation; however, it was not possible to estimate edible food waste.

Finally, it should be noted that there are uncertainties related to climate impact data, e.g., climate impact for a given food type can vary according to the production system for different regions and depending on the choice of database. The Cool Food Pledge Calculator includes default weighted regional average data for GHGE [57] and thereby does not represent specific Danish values. Moreover, GHGE values from Metric 4 (global-average factors for carbon opportunity costs) may be overestimated compared with other estimates that include land-use change (LUC) [28]. Methods of estimating the LUC are still under discussion [58], and carbon opportunity costs based on the values from Searchinger et al. 2018 are high compared to other values [59]. Thus, the GHGE assessment reflects an approximation, and the focus should be on the estimated reduction rather than absolute emission vales.

## 5. Conclusions

The present study represents an important step in shifting public food procurement, including food for nursing homes, in a more climate-friendly direction while at the same time improving the nutritional quality of the served meals. The guidance can be adapted for use by other municipalities, hospitals and catering services providing food to older frail adults. Compared with current practices, major changes included a decrease in serving amounts of meat to no more than 500 g cooked meat per week and an increase in plant-based protein-rich sources such as pulses, nuts and seeds. Moreover, animal-based fat sources were decreased in favour of more plant-based fat sources. The guidelines for menu planning should be supported by activities that help the kitchen staff in adoption of the diets. Further, the diets should be tested in a real-life environment among older adults living in nursing homes in order to explore the acceptability of the guidelines and the effects on dietary intake and health outcomes. This kind of innovation in food procurement is required in order to achieve the necessary transition to a healthy and sustainable food system.

## Figures and Tables

**Table 1:** Content of foods per 10 MJ at baseline based on the municipality’s procurement data for nursing homes with in-house meal production and for an energy- and protein-dense diet (Scenario 1), a standard protein-dense diet, (Scenario 2) and a combined scenario reflecting nursing homes serving a combination of diets (Scenario 3). Changes compared to baseline are shown in parenthesis.

	Baseline: Nursing Homes (g per 10 MJ)	Scenario 1: Energy- and Protein-Dense Diet (g per 10 MJ)	Scenario 2: Standard Protein-Dense Diet (g per 10 MJ)	Scenario 3: Combined Diet (g per 10 MJ)
Bread, grains and cereals	142	106	218	140 (−2%)
Potatoes	122	50	122	71 (−41%)
Vegetables	129	70	250	124 (−4%)
Mushrooms	5	3	5	4 (−23%)
Pulses, whole (dried weight) ^1^	2	26 (20)	26 (20)	26 (20) (+1334%)
Pulses, flour/protein powder	3	45	15	36 (+1711%)
Nuts	1	20	20	20 (+1583%)
Seeds ^2^	1	16	16	16 (+1113%)
Fruits ^3^	108	60	250	117 (+8%)
Dried fruit	1	1	1	1 (0%)
Milk (liquid) ^3^	248	248	248	248 (0%)
Yoghurt products	82	100	82	95 (+16%)
Cream and crème fraiche	71	45	5	33 (−53%)
Cheese	34	23	23	23 (−33%)
Beef and lamb ^4,5^	36	16	16	16 (−56%)
Pork ^5^	71	62	62	61 (−13%)
Poultry ^5^	19	19	19	19 (0%)
Eggs	34	38	38	38 (+10%)
Fish, total ^5^	44	61	61	61 (+37%)
Fats ^6^, plant based	23	30	11	25 (+5%)
Fats ^6^, animal based	33	20	10	17 (−48%)
Coffee, tea	1	1	1	1 (0%)
Discretionary foods/beverages	79	56	31	48 (−39%)
Condiments and seasoning	20	20	20	20 (0%)

^1^ Mixture of dried and processed/cooked pulses (conversion to dried amount shown in parenthesis); ^2^ furthermore, the bread contained 1–2 g seeds; ^3^ whole milk for the energy- and protein-dense menu and low-fat milk (“keyhole-labelled”) for the standard protein-dense diet; ^4^ includes small amount of beef in mixed beef and pork products; ^5^ includes mainly raw meat/fish; ^6^ Includes fatty products, such as fatty sauces and dressings.

**Table 2:** Content of macronutrients at the baseline based on a municipality’s procurement data for nursing homes with in-house food production and in different scenarios: an energy- and protein-dense diet (Scenario 1), a standard protein-dense diet (Scenario 2) and a combined scenario reflecting nursing homes serving a combination of diets (Scenario 3).

Nutrients ^1^	Baseline: Nursing Homes	Scenario 1: Energy- and Protein-Dense Diet	Scenario 2: Standard Protein-Dense Diet	Scenario 3: Combined Diet	Recommended Macronutrient Density ^1^
Protein, total, g per 10 MJ	75	103	103	103	
Protein, total, E%	13	18	18	18	18
Carbohydrates, g per 10 MJ	226	182	277	210	
Carbohydrates, E%	40	33	50	38	
Added sugar, E%	12	8	7	8	
Fat total, g per 10 MJ	128	135	88	121	
Fat total, E%	47	33	50	45	50/32–33
Saturated fatty acids, E%	21	17	10	15	
n-3 fatty acids, E%	1.3	1.8	1.3	1.6	
Dietary fibre, g per 10 MJ	18	23	38	27	

^1^ Recommended nutrient density according to the Danish official recommendations regarding the food served in Danish institutions for an energy- and protein-dense diet [14] and for planning diets for groups of individuals (+65 y) (standard protein-dense diet) from NNR [16].

**Table 3:** Guidelines for menu planning, including rules of thumb for serving frequency and estimated average serving amounts per week per nursing home resident having an energy requirement of 9 MJ/d (2.100 kcal/d). The guidelines are the same for the energy- and protein-dense diet and the standard protein-dense diet, unless otherwise stated.

Guidelines for Menu Planning ^1^	Weekly Average Serving Amounts ^2^
1. Serve meat in moderate quantities and rarely beef, veal and lamb	
Hot meal: Serve meat max 5 times a week (average serving size 75 g) including beef, veal or lamb max once a week. Cold main meal: Serve max one type of meat per meal as cold cuts/spreads on bread. Beef, veal and lamb can be served once every second week.	500 g cooked meat including no more than 80–85 g beef, veal and lamb
2. Serve fish and choose the most sustainable	
Hot meal: Serve fish at least once a week (average serving size 100–125 g). Cold main meal: Serve fish every day as cold cuts/spreads on bread.	300 g cooked fish
3. Serve pulses daily in both main meals and in-between meals	
Hot meal: Use pulses/pulse flour 4–5 times a week ^3^, e.g., together with meat, blended in soup, sauce, in bread etc. Other meals: Use pulses/pulse flour every day in the cold main meal (e.g., part of bread or as filling) and in the in-between meals (e.g., in cakes). In addition, use in energy- and protein-dense drinks for the energy- and protein-dense diet.	300 g cooked pulses ^3^. In addition, 300 g pulse flour for the energy- and protein-dense diet and 100 g pulse flour for the standard protein-dense diet ^3^
4. Use nuts and seeds daily	
Hot meal: Use nuts ^4^, e.g., 2–3 times a week and seeds, e.g., twice a week. Other meals: Use nuts, e.g., 3–4 times a week and seeds, e.g., every day in small amounts in, e.g., bread, cakes, filling etc.	125 g nuts and 100 g seeds ^5^
5. Use eggs and cheese in moderate quantities and prefer protein-rich dairy products	
Hot meal: Use eggs once a week as a main component (e.g., in an omelet). Use eggs and cheese in smaller amounts in, e.g., tarts, desserts etc. Prefer protein-rich dairy products. Other meals: Use egg and protein-rich dairy products daily at breakfast, in the cold meal or in the in-between meals. Use cheese once a day. Use fatty dairy products 1–2 times a day in the energy- and protein-dense diet.	240 g egg, 140 g cheese and 550 g protein-rich dairy products. In addition, 275 g fatty dairy products for the energy- and protein-dense diet
6. Use plant-based fats often and limit animal fats	
Hot meal: Use mainly plant-based fats. Use animal fats such as butter no more than twice a week. Other meals: Use mainly plant-based fats. Use animal fats such as butter no more than 1–2 times a day.	325 g fats (including 200 g plant-based fats) for the energy- and protein-dense diet and 125 g fats (including 70 g plant-based fats) for the standard protein-dense diet
7. Serve vegetables/fruits in all meals and in many colours	
Hot meal: Serve fruits and vegetables in all meals including both dark green and red-orange vegetables several times a week. Other meals: Serve fruits and vegetables in all main meals.	Approx. 700 g for the energy- and protein-dense diet and approx. 3 kg for the standard protein-dense diet. In addition, juice
8. Serve potatoes and both whole grain and non-whole grain cereals	
Hot meal: Serve starchy foods at all meals depending on taste preferences, e.g., potatoes 4–5 times a week. Other meals: About half of the grain products in the standard protein-dense diet should be whole grain products.	Approx. 1.1 kg for the energy- and protein-dense diet and approx. 2½ kg for the standard protein-dense diet
9. Offer drinking milk and water daily	
Offer up to two glasses of milk every day.	225 g per day ^6^. Offer whole-fat and low-fat milk for the energy- and protein-dense and energy- and protein-dense diet, respectively
10. Follow the season and limit food waste	

^1^ An accompanying text should emphasize that meals should be palatable and recognizable as well as the need to focus on texture-modified meals for residents with swallowing and/or chewing disorders; ^2^ amounts must be adjusted according to the residents’ appetite and preferences; ^3^ pulses and pulse flour can partly be exchanged with other plant-based protein-rich foods to add variation, including processed soy-based products such as tofu or meat-replacers. Likewise, pulse flour may be partly exchanged with other plant-based protein-rich flours and powders and in smaller amounts with animal-based powders; ^4^ includes groundnuts. Salt content of nuts max 0.8 g/100 g; ^5^ includes seeds in bread; ^6^ drinking milk can be substituted with milk/dairy for, e.g., breakfast cereals.

**Table 4:** Estimated GHGE from total agricultural supply chains (Metric 2) and combined total agricultural supply chains and carbon opportunity costs (COC) (Metric 2 + 4) [29] at the baseline purchase and for the combined diet (Scenario 3).

Metrics	Baseline: Nursing Homes ^1^ (per 10 MJ)	Scenario 3: Combined Diet (per 10 MJ)
GHGE (Metric 2) (kg CO_2_-e)	5.2	4.1 (−22%)
GHGE inclusive COC (Metric 2 + 4) (kg CO_2_-e)	22.7	16.9 (−26%)

^1^ Nursing homes with in-house meal production.

## Data Availability

The data that support the findings of this study were provided by the City of Copenhagen, but restrictions apply to the availability of these data, which were used with permission for the current study and thus are not publicly available. In case of further interest in these data, requests must be made to the City of Copenhagen’s Budget Team in the Children and Youth Administration.

## References

[1] Crippa M., Solazzo E., Guizzardi D., Monforti-Ferrario F., Tubiello F.N., Leip A. (2021). Food systems are responsible for a third of global anthropogenic GHG emissions. Nat. Food.

[2] Willett W., Rockström J., Loken B., Springmann M., Lang T., Vermeulen S., Garnett T., Tilman D., DeClerck F., Wood A. (2019). Food in the Anthropocene: The EAT-Lancet Commission on healthy diets from sustainable food systems. Lancet.

[3] Rose D., Heller M.C., Roberto C.A. (2019). Position of the Society for Nutrition Education and Behavior: The Importance of Including Environmental Sustainability in Dietary Guidance. J. Nutr. Educ. Behav..

[4] Boyano A., Espinosa N., Rodriguez Quintero R., Neto B., Gama Caldas M., Wolf O. (2019). EU GPP Criteria for Food Procurement, Catering Services and Vending Machines.

[5] United Nations Environment Programme: Global Review of Sustainable Public Procurement 2017. https://www.oneplanetnetwork.org/sites/default/files/globalreview_web_final.pdf.

[6] World Health Organization (2021). Action Framework for Developing and Implementing Public Food Procurement and Service Policies for a Healthy Diet.

[7] Jörgensen C. (2021). Stimulating Green Production through the Public Procurement of Final Products—The Case of Organic Food, AgriFood-WP 2021:2.

[8] Global Lead City Network on Sustainable Procurement (2021). Food & Catering. Global Public Procurement Factsheet.

[9] The City of Copenhagen (2019). The City of Copenhagen’s Food Strategy [in Danish: Københavns kommunes Mad- og Måltidsstrategi 2019].

[10] Beck A.M., Dent E., Baldwin C. (2016). Nutritional intervention as part of functional rehabilitation in older people with reduced functional ability: A systematic review and meta-analysis of randomised controlled studies. J. Hum. Nutr. Diet..

[11] Rodríguez-Rejón A.I., Ruiz-López M.D., Malafarina V., Puerta A., Zuniga A., Artacho R. (2017). Menus offered in long-term care homes: Quality of meal service and nutritional analysis. Nutr. Hosp..

[12] Volkert D., Beck A.M., Cederholm T., Cruz-Jentoft A., Goisser S., Hooper L., Kiesswetter E., Maggio M., Raynaud-Simon A., Sieber C.C. (2019). ESPEN guideline on clinical nutrition and hydration in geriatrics. Clin. Nutr..

[13] Okkels S.L., Saxosen M., Bügel S., Olsen A., Klausen T.W., Beck A.M. (2018). Acceptance of texture-modified in-between-meals among old adults with dysphagia. Clin. Nutr. Espen.

[14] Pedersen A.N., Ovesen L. (2015). Recommendations of the Danish Institution Diet, 1st ed [in Danish].

[15] Arjuna T., Miller M., Ueno T., Visvanathan R., Lange K., Soenen S., Chapman I., Luscombe-Marsh N. (2018). Food services using energy- and protein-fortified meals to assist vulnerable community-residing older adults meet their dietary requirements and maintain good health and quality of life: Findings from a pilot study. Geriatrics.

[16] Nordic Council of Ministers (2014). Nordic Nutrition Recommendations 2012. Integrating Nutrition and Physical Activity.

[17] Bauer J., Biolo G., Cederholm T., Cesari M., Cruz-Jentoft A.J., Morley J.E., Phillips S., Sieber C., Stehle P., Teta D. (2013). Evidence-based Recommendations for Optimal Dietary Protein Intake in Older People: A Position Paper From the PROT-AGE Study Group. J. Am. Med Dir. Assoc..

[18] Beck A.M., Ovesen L. (2002). Body mass index, weight loss and energy intake of old Danish nursing home residents and home-care clients. Scand. J. Caring Sci..

[19] Beck A.M. (2012). The relationship between weight status and the need for health care assistance in nursing home residents. J. Aging Res. Clin. Pract..

[20] Boscart V., Crutchlow L.E., Sheiban Taucar L., Johnson K., Heyer M., Davey M., Costa A.P., Heckman G. (2020). Chronic disease management models in nursing homes: A scoping review. BMJ Open.

[21] Broeckhoven I., Verbeke W., Tur-Cardona J., Speelman S., Hung Y. (2021). Consumer valuation of carbon labeled protein-enriched burgers in European older adults. Food Qual. Prefer..

[22] Okkels S.L., Dybdal D.R., Pedersen R.J., Klausen T.W., Olsen A., Beck A.M., Bügel S. (2021). A culinary twist of a two-course meals-on-wheels menu in a cluster-randomized controlled trial influencing health-related quality of life in nursing home residents. Clin. Nutr. Espen.

[23] Saxe H., Jensen J.D., Bølling Laugesen S.M., Bredie W.L.P. (2018). Environmental impact of meal service catering for dependent senior citizens in Danish municipalities. Int. J. Life Cycle Assess..

[24] Verzola D., Picciotto D., Saio M., Aimasso F., Bruzzone F., Sukkar S.G., Massarino F., Esposito P., Viazzi F., Garibotto G. (2021). Low protein diets and plant-based low protein diets: Do they meet protein requirements of patients with chronic kidney disease?. Nutrients.

[25] Hansen K.V., Derdowski L.A. (2020). Sustainable food consumption in nursing homes: Less food waste with the right plate color?. Sustainability.

[26] Trolle E., Thorsen A.V., Tørsleff E.H., Bernhards-Werge J., Lassen A.D. (2019). Food in Elderly Care Centers—Connection between the Use of Organic Products and the Nutritional Quality of Food as well as the Degree of Processing When Shopping [In Danish: Mad på Plejecentre—Sammenhæng Mellem Brug af Økologiske Produkter og den Ernæringsmæssige Kvalitet af Maden samt Forarbejdningsgrad ved Indkøb].

[27] DTU Food (2019). Food Data, Version 4. Frida.fooddata.dk.

[28] Lassen A.D., Nordman M., Christensen L.M., Trolle E. (2021). Scenario Analysis of a Municipality’s Food Purchase to Simultaneously Improve Nutritional Quality and Lower Carbon Emission for Child-Care Centers. Sustainability.

[29] World Resources Institute The Cool Food Pledge. https://www.wri.org/our-work/project/cool-food-pledge.

[30] Didinger C., Thompson H.J. (2021). Defining nutritional and functional niches of legumes: A call for clarity to distinguish a future role for pulses in the dietary guidelines for americans. Nutrients.

[31] The Danish Diet and Nutrition Association (2021). The Dietary Reference Manual. https://xn--kosthndbogen-xcb.dk/.

[32] Cereda E., Pedrolli C., Klersy C., Bonardi C., Quarleri L., Cappello S., Turri A., Rondanelli M., Caccialanza R. (2016). Nutritional status in older persons according to healthcare setting: A systematic review and meta-analysis of prevalence data using MNA. Clin. Nutr..

[33] Beck A.M., Hansen K.S. (2010). Meals served in Danish nursing homes and to meals-on-wheels clients may not offer nutritionally adequate choices. J. Nutr. Elder..

[34] Iuliano S., Poon S., Wang X., Bui M., Seeman E. (2017). Dairy food supplementation may reduce malnutrition risk in institutionalised elderly. Br. J. Nutr..

[35] Woods J.L., Walker K.Z., Iuliano-Burns S., Strauss B.J. (2009). Malnutrition on the menu: Nutritional status of institutionalised elderly Australians in low-level care. J. Nutr. Health Aging.

[36] National Nutrition Council, Finnish Institute for Health and Welfare (2020). Vitality in Later Years—Food Recommendation for Older Adults. Directions 9/2020.

[37] Danish Veterinary and Food Administration (2021). The Official Dietary Guidelines—Good for Health and Climate. Glostrup, Ministry of Food, Agriculture and Fisheries.

[38] Danish Veterinary and Food Administration (2021). Advice on Food and Drink When You Are Over 65 Years Old (In Danish). Glostrup, Ministry of Food, Agriculture and Fisheries.

[39] Dominguez L.J., Veronese N., Barbagallo M. (2021). Magnesium and hypertension in old age. Nutrients.

[40] Cruz-Jentoft A.J., Calvo J.J., Durán J.C., Ordénez J., de Castellar R. (2008). Compliance with an oral hyperproteic supplement with fibre in nursing home residents. J. Nutr. Health Aging.

[41] Guyomarc’h F., Arvisenet G., Bouhallab S., Canon F., Deutsch S.M., Drigon V., Duponta D., Famelarta M.-H., Garrica G., Guédon E. (2021). Mixing milk, egg and plant resources to obtain safe and tasty foods with environmental and health benefits. Trends Food Sci. Technol..

[42] Liu J., Klebach M., Visser M., Hofman Z. (2019). Amino acid availability of a dairy and vegetable protein blend compared to single casein, whey, soy, and pea proteins: A double-blind, cross-over trial. Nutrients.

[43] Francis Raguindin P., dam Itodo O., Stoyanov J., Dejanovic G.M., Gamba M., Asllanaj E., Mindere B., Busslerf W., Metzgerf B., Muka T. (2021). A systematic review of phytochemicals in oat and buckwheat. Food Chem..

[44] Beelen J., de Roos N.M., de Groot L.C.P.G. (2017). Protein enrichment of familiar foods as an innovative strategy to increase protein intake in institutionalized elderly. J. Nutr. Health Aging.

[45] Svendsen J.A., Okkels S.L., Knudsen A.W., Munk T., Beck A.M. (2021). Sensory acceptance of food developed for older adults in different settings. J. Sens. Stud..

[46] Pintado T., Delgado-Pando G. (2020). Towards more sustainable meat products: Extenders as a way of reducing meat content. Foods.

[47] Argel N.S., Ranalli N., Califano A.N., Andrés S.C. (2020). Influence of partial pork meat replacement by pulse flour on physicochemical and sensory characteristics of low-fat burgers. J. Sci. Food Agric..

[48] Boukid F., Zannini E., Carini E., Vittadini E. (2019). Pulses for bread fortification: A necessity or a choice?. Trends Food Sci. Technol..

[49] Binou P., Yanni A.E., Karathanos V.T. (2020). Physical properties, sensory acceptance, postprandial glycemic response, and satiety of cereal based foods enriched with legume flours: A review. Crit. Rev. Food Sci. Nutr..

[50] Thompson H.J. (2019). Improving human dietary choices through understanding of the tolerance and toxicity of pulse crop constituents. Curr. Opin. Food Sci..

[51] Weijzen M.E.G., Kouw I.W.K., Geerlings P., Verdijk L.B., van Loon L.J.C. (2020). During Hospitalization, Older Patients at Risk for Malnutrition Consume <0.65 Grams of Protein per Kilogram Body Weight per Day. Nutr. Clin. Pract..

[52] Paddon-Jones D., Rasmussen B.B. (2009). Dietary protein recommendations and the prevention of sarcopenia. Curr. Opin. Clin. Nutr. Metab. Care.

[53] Wendin K., Biorklund-Helgesson M., Andersson-Stefanovic K., Lareke A., Book O., Skjoldebrand C. (2021). Liking, preference and practical implications of protein and energy enriched in-between-meals designed for elderly people. Food Nutr. Res..

[54] Roberts H.C., Lim S.E.R., Cox N.J., Ibrahim K. (2019). The challenge of managing undernutrition in older people with frailty. Nutrients.

[55] Moore D.R., Churchward-Venne T.A., Witard O., Breen L., Burd N.A., Tipton K.D., Phillips S.M. (2015). Protein ingestion to stimulate myofibrillar protein synthesis requires greater relative protein intakes in healthy older versus younger men. J. Gerontol. Ser. A Biol. Sci. Med Sci..

[56] Tieva Å., Persson E., Rhodin A., Sköldunger A., Pettersén S., Jonsäll A., Hörnell A. (2015). Effect on energy and macronutrient intake with partial replacement of external food supply by in-house cooking at a nursing home for older people in Sweden. Int. J. Consum. Stud..

[57] Waite R., Vennard D., Pozzi G. (2020). Tracking Progress towards the Cool Food Pledge: Setting Climate Targets, Tracking Metrics, Using the Cool Food Calculator, and Related Guidance for Pledge Signatories.

[58] Zampori L., Pant R. (2019). Suggestions for Updating the Product Environmental Footprint (PEF) Method, EUR 29682 EN.

[59] Searchinger T.D., Wirsenius S., Beringer T., Dumas P. (2018). Assessing the efficiency of changes in land use for mitigating climate change. Nature.

